# The Neurokinin-1 Receptor Contributes to the Early Phase of Lipopolysaccharide-Induced Fever *via* Stimulation of Peripheral Cyclooxygenase-2 Protein Expression in Mice

**DOI:** 10.3389/fimmu.2018.00166

**Published:** 2018-02-05

**Authors:** Eszter Pakai, Valeria Tekus, Csaba Zsiboras, Zoltan Rumbus, Emoke Olah, Patrik Keringer, Nora Khidhir, Robert Matics, Laszlo Deres, Katalin Ordog, Nikolett Szentes, Krisztina Pohoczky, Agnes Kemeny, Peter Hegyi, Erika Pinter, Andras Garami

**Affiliations:** ^1^Institute for Translational Medicine, Medical School, University of Pecs, Pecs, Hungary; ^2^Momentum Gastroenterology Multidisciplinary Research Group, Hungarian Academy of Sciences – University of Szeged, Szeged, Hungary; ^3^Department of Pharmacology and Pharmacotherapy, Medical School, University of Pecs, Pecs, Hungary; ^4^Janos Szentagothai Research Centre, University of Pecs, Pecs, Hungary; ^5^First Department of Medicine, Medical School, University of Pecs, Pecs, Hungary; ^6^Department of Medical Biology, Medical School, University of Pecs, Pecs, Hungary; ^7^First Department of Medicine, University of Szeged, Szeged, Hungary

**Keywords:** fever, thermoregulation, systemic inflammation, endotoxin, cyclooxygenase, autonomic thermoeffectors, substance P, *Tacr1*

## Abstract

Neurokinin (NK) signaling is involved in various inflammatory processes. A common manifestation of systemic inflammation is fever, which is usually induced in animal models with the administration of bacterial lipopolysaccharide (LPS). A role for the NK1 receptor was shown in LPS-induced fever, but the underlying mechanisms of how the NK1 receptor contributes to febrile response, especially in the early phase, have remained unknown. We administered LPS (120 µg/kg, intraperitoneally) to mice with the *Tacr1* gene, i.e., the gene encoding the NK1 receptor, either present (*Tacr1^+/+^*) or absent (*Tacr1^−/−^*) and measured their thermoregulatory responses, serum cytokine levels, tissue cyclooxygenase-2 (COX-2) expression, and prostaglandin (PG) E_2_ concentration. We found that the LPS-induced febrile response was attenuated in *Tacr1^−/−^* compared to their *Tacr1^+/+^* littermates starting from 40 min postinfusion. The febrigenic effect of intracerebroventricularly administered PGE_2_ was not suppressed in the *Tacr1^−/−^* mice. Serum concentration of pyrogenic cytokines did not differ between *Tacr1^−/−^* and *Tacr1^+/+^* at 40 min post-LPS infusion. Administration of LPS resulted in amplification of COX-2 mRNA expression in the lungs, liver, and brain of the mice, which was statistically indistinguishable between the genotypes. In contrast, the LPS-induced augmentation of COX-2 protein expression was attenuated in the lungs and tended to be suppressed in the liver of *Tacr1^−/−^* mice compared with *Tacr1^+/+^* mice. The *Tacr1^+/+^* mice responded to LPS with a significant surge of PGE_2_ production in the lungs, whereas *Tacr1^−/−^* mice did not. In conclusion, the NK1 receptor is necessary for normal fever genesis. Our results suggest that the NK1 receptor contributes to the early phase of LPS-induced fever by enhancing COX-2 protein expression in the periphery. These findings advance the understanding of the crosstalk between NK signaling and the “cytokine-COX-2-prostaglandin E_2_” axis in systemic inflammation, thereby open up the possibilities for new therapeutic approaches.

## Introduction

The neurokinin-1 (NK1) receptor, formerly also known as substance P (SP) receptor, plays an important role in mediation of local and systemic inflammatory processes (1). As part of systemic inflammation the most often developing thermoregulatory response is fever, which is commonly induced in experimental animals by the administration of bacterial lipopolysaccharide (LPS). In the development of the febrile response to LPS several molecular mechanisms have been already identified (2, 3). In brief, LPS triggers the activation of peripheral macrophages, which then produce inflammatory cytokines such as interleukin (IL)-1β, IL-6, and tumor necrosis factor (TNF)-α and induce the activation of the arachidonic acid cascade. Arachidonic acid is produced from membrane phospholipids by the action of phospholipases A_2_ [PLA_2_; reviewed in Ref. (4)] and *via* alternative pathways, such as monoacylglycerol lipase-dependent hydrolysis (5). In the next step of febrigenesis, the cyclooxygenase-2 (COX-2) enzyme is of crucial importance as it has been shown that selective blockade of COX-2 completely abolishes the fever response (6, 7). Among the end products of the cascade, prostaglandin (PG) E_2_ is synthesized by terminal PGE synthases, which can be microsomal and cytosolic (8). PGE_2_ is a key mediator, since it is produced in the periphery already in the early stage of fever (9) and because its binding to EP3 receptors in the hypothalamus triggers the activation of thermogenesis and cutaneous vasoconstriction, thereby resulting in fever (10, 11). In addition to the aforementioned mechanisms, various further substances have been identified as mediators of fever, which also include SP signaling (3, 12).

Indicating the role of SP in fever, when the effects of SP were antagonized with peptide SP analogs, the fever response to LPS was blocked in guinea pigs (13) and in rats (14). Similar attenuation of the LPS-induced fever was observed in rats after administration of the NK1 receptor antagonists CP-96,345 (15) and SR140333B (16). These studies strongly support that SP signaling contributes to the development of LPS-induced fever, but it has remained largely unknown which mediators of the febrile process are influenced by SP or its receptors. In all of these studies, the authors used antagonists, which can be problematic because of their short half-lives, poor brain penetration, and off-target effects (17). In an earlier study, SP inhibited pancreatic bicarbonate secretion *via* NK2 and NK3 receptors (18), suggesting that the effects of SP or its peptide analog antagonists are not solely mediated by NK1 receptors. In the case of the non-peptide antagonists, it was shown that at higher doses CP-96,345 and SR140333B also block L-type calcium channels (19, 20). Alternative approaches, such as the use of knockout mice, can help to complement the findings with antagonists about the contribution of the NK1 receptor to fever. In addition to complementing the earlier findings with antagonists, by using knockout mice our main goal was to better clarify which step(s) of the classical molecular mechanisms of fever are influenced by the NK1 receptor.

In the present work, we studied how genetic ablation of the NK1 receptor influences the LPS-induced fever response in mice. In thermophysiological experiments, we recorded changes in deep body temperature (*T*_b_) and in the activity of autonomic thermoeffectors in response to LPS and PGE_2_. To identify the involved molecular mechanisms, we measured serum cytokine levels, as well as tissue COX-2 mRNA and protein expression, and PGE_2_ concentration in the same animal model.

## Materials and Methods

### Animals

The experiments were performed in 174 adult mice of both sexes. To minimize the possibility that gender of the mice has an influence on our results, male and female mice were approximately equally distributed in age-matched experimental groups. Of the mice, 86 had the *Tacr1* gene, i.e., the gene encoding the NK1 receptor, homozygously present (*Tacr1^+/+^*), while 88 absent (*Tacr1^−/−^*) due to a targeted disruption (21). The *Tacr1^−/−^* mice were generated at the University of Liverpool as described in detail elsewhere (21). The original breeding pairs of the *Tacr1^−/−^* mice were donated to the University of Pecs by Dr. John Quinn (University of Liverpool). Their breeding and backcrossing on a C57BL/6 background (for at least 10 generations) were reported in our recent study (22). The mice were housed in standard plastic cages kept in a room with an ambient temperature maintained at 25–27°C and with a humidity of 30–40%. The room was on a 12 h light–dark cycle (lights on at 5:00 a.m.). Standard rodent chow and tap water were available *ad libitum*. At the time of the experiments, the *Tacr1^+/+^* and *Tacr1^−/−^* mice weighed 21 ± 2 and 19 ± 2 g, respectively.

The mice were extensively handled and then habituated to staying inside wire-mesh cylindrical confiners. The cylindrical confiner prevented the animal from turning around, but allowed for some back-and-forth movements; it was used in the thermocouple and respirometry setups (see Experimental Setups).

All procedures were conducted under protocols approved by Institutional Animal Use and Care Committee of the University of Pecs and were in accordance with the directives of the National Ethical Council for Animal Research and those of the European Communities Council (86/609/EEC).

### Surgeries

Mice were anesthetized with the intraperitoneal (i.p.) administration of a ketamine–xylazine cocktail (81.7 and 9.3 mg/kg, respectively) and received antibiotic protection intramuscularly (gentamycin, 6 mg/kg). During surgery, a mouse was heated with a temperature-controlled heating pad (model TMP-5a; Supertech Instruments UK Ltd., London, UK) placed under a surgery board. For pain management, ketoprofen (5 mg/kg) was administered subcutaneously at the end of surgery and on the next day. The experiments were performed 4–7 days after the surgery.

For i.p. catheter implantation, a small midline incision was made on the abdominal wall, and then a polyethylene (PE)-50 catheter filled with pyrogen-free saline was inserted into the peritoneal cavity. The internal end of the catheter was fixed to the left side of the abdominal wall with a suture; the free end of the catheter was tunneled under the skin to the nape where it was exteriorized and heat-sealed. The surgical wound was sutured in layers. The catheter was flushed with 0.1 ml of saline on the day after the surgery and every other day thereafter. No sign of discomfort or inflammation was associated with this procedure in the present study and in our other studies in mice (23, 24) and in rats (24, 25).

For intracerebroventricular (i.c.v.) cannula implantation, each mouse was fixed to a stereotaxic apparatus similarly as in our earlier study (26). The scalp was incised over the sagittal suture; the periosteum was excised; the skull was cleaned and dried; two supporting microscrews were driven into the skull; and a small hole was drilled in the skull 0.5 mm posterior from bregma and 1.0 mm lateral from midline. A 22-G steel guide cannula (Plastics One, Roanoke, VA, USA) was attached to a plastic tube fitted into a stereotaxic manipulator (Narishige Scientific Instruments Laboratory, Tokyo, Japan), which was used to insert the cannula into the brain through the bone hole. The tip of the cannula was placed within the right lateral ventricle (2.0 mm from dura). The cannula was secured to the supporting microscrews with dental cement and released from the manipulator. The guide cannula was closed by a dummy cannula.

### Experimental Setups

The thermophysiological experiments were performed in the thermocouple or in the respirometry setup.

In the thermocouple setup, the mouse was placed in a cylindrical confiner and equipped with copper-constantan thermocouples (Omega Engineering, Stamford, CT, USA) to measure colonic temperature (a form of deep *T*_b_) and tail skin temperature (*T*_sk_). The colonic thermocouple was inserted 3 cm deep beyond the anal sphincter and was fixed to the base of the tail with a loop of adhesive tape. The skin thermocouple was positioned on the lateral surface of tail at the border between the proximal and middle third of the tail and secured in place with tape. The thermocouples were plugged into a data logger device (Cole-Palmer, Vernon Hills, IL, USA) connected to a computer. Mice in their confiners were then placed into a temperature-controlled incubator (model MIDI F230S; PL Maschine Ltd., Tarnok, Hungary) set to an ambient temperature of 31°C, which is at the lower end of the thermoneutral zone for mice in this setup. When present, the i.p. catheter was connected to a PE-50 extension filled with the drug of interest. When the mouse had an i.c.v. cannula, a needle injector was fitted into the guide cannula and connected to a PE-50 extension. The extension was passed through a port of the chamber and connected to a syringe. In a separate set of experiments, the mice were exposed to heat or cold by applying similar protocols as in our earlier studies (26, 27). The mice in their confiners were placed into a biochemistry incubator (model BJPX-Newark; Biobase, Jinan, China) initially set to an ambient temperature of 33°C, which was then either raised to 39°C or decreased to 15°C over ~30 min and maintained at 39 or 15°C to expose the mice to heat or cold, respectively.

A mouse designated for an experiment in the respirometry setup was equipped with thermocouples and placed in a confiner as in experiments in the thermocouple setup. Then, the mouse in its confiner was transferred to a Plexiglas chamber of the four-chamber open-circuit calorimeter integrated system (Oxymax Equal Flow, Columbus Instruments), as in our earlier studies (25, 27). The chamber was sealed, submerged into a temperature-controlled (31°C) water bath, and continuously ventilated with room air (200 ml/min). The fractional concentration of oxygen was measured in the air entering and exiting the chamber, and the rate of oxygen consumption (VO_2_) was calculated according to the manufacturer’s instructions using the Oxymax Windows software (version 3.1). The extension of the i.p. catheter or the i.c.v. needle injector was passed through a port of the chamber and connected to a syringe, which was placed in a syringe pump (model 975; Harvard Apparatus Inc., Holliston, MA, USA).

### Substance Administration

Lipopolysaccharide from *Escherichia coli* 0111:B4 was purchased from Sigma-Aldrich (St. Louis, MO, USA). A stock suspension of LPS (5 mg/ml) in pyrogen-free saline was stored at −20°C. On the day of the experiment, the stock was diluted to a final concentration of 36 µg/ml. The diluted LPS suspension or saline was infused (26 µl/min for 4 min) through the extension of the i.p. catheter to deliver LPS at a final dose of 120 µg/kg. Deep *T*_b_, tail *T*_sk_, and VO_2_ were monitored for 6 h after the injection.

Substance P and PGE_2_ were purchased from Tocris Bioscience (Bristol, UK). Aliquots of ethanolic stock solutions of SP and PGE_2_ (10 and 12.5 mg/ml, respectively) were stored at −80°C. On the day of the experiment, the stock solutions were diluted with ethanol and saline to give working solutions of SP and PGE_2_ (1,000 and 33 µg/ml, respectively) in 10% ethanol. By infusing these solutions into the lateral ventricle (1 µl/min for 3 min), a total dose of either 100 µg/kg SP or 3.3 µg/kg PGE_2_ was delivered i.c.v. Control mice were infused with the vehicle (10% ethanol in saline). For the infusion, the dummy injector was removed from the preimplanted guide cannula and replaced with a 28-G injector needle (Plastics One) connected to a 10-µl Hamilton syringe by a PE-50 extension. The injector needle protruded 1.0 mm beyond the tip of the guide cannula.

Administration of the substances was carried out between 10:30 a.m. and 12:00 p.m. in the experiments.

### Molecular Biology

#### Tissue Harvesting

Each mouse was implanted with an i.p. catheter and extensively adapted to the experimental setup. On the day of experiment, each mouse was placed in a confiner and transferred to an incubator chamber, which was set to an ambient temperature of 31°C. The i.p. catheter was connected to a PE-50 extension filled with LPS or saline. The extension was passed through a port of the chamber and connected to a syringe, which was placed in a syringe pump (Harvard Apparatus Inc.). Mice were left to acclimate for ~2 h and then infused with LPS or saline as in the thermophysiological experiments. Forty minutes after infusion, the mice were anesthetized with ketamine–xylazine cocktail, which was injected through the extension of the i.p. catheter. Blood samples were collected from the left ventricle. Each sample was transferred to an ice-cold Eppendorf tube containing EDTA (40 µl) and trasylol (20 µl). The collected blood was immediately centrifuged at 1,000 rpm for 5 min, then at 4,000 rpm for 10 min, and the resulting plasma was stored at −80°C. For collection of lung, liver, and brain tissue samples for RT-qPCR, Western blot, and immunoassay protocols, each mouse was perfused through the left ventricle with 0.1 M phosphate-buffered saline. Samples of the liver and the right lung were collected rapidly and snap frozen in liquid nitrogen. The anesthetized mouse was decapitated, its entire brain was removed and frozen. All tissue samples were stored at −80°C.

#### Immunoassays

The serum level of TNF-α was determined with enzyme-linked immunosorbent assay (ELISA) by using a commercially available mouse TNF ELISA kit (BD OptEIA catalog nr: 560478; BD Biosciences, San Jose, CA, USA), which had a detection limit of 31 pg/ml for TNF-α. PGE_2_ concentrations in the lungs, liver, and brain were measured by ELISA using a commercially available kit (catalog nr: 514010; Cayman Chemical, Ann Arbor, MI, USA); the samples were prepared according to the manufacturer’s instructions. The assay had a sensitivity of 15 pg/ml for PGE_2_. Detections were performed by using the Labsystem Multiskan RC plate reader (Thermo Scientific, Waltham, MA, USA), as in our earlier study (22).

Serum concentrations of granulocyte-macrophage colony-stimulating factor (GM-CSF) and IL-6 were determined by Luminex’s xMAP^®^ Technology using a multiplex bead immunoassay kit (catalog nr: LMC0003; Invitrogen, Carlsbad, CA, USA). After thawing, serum samples were centrifuged (1,000 *g* for 10 min) to prevent clogging of the filter plate. The measurement was performed according to the manufacturer’s instructions. Following previous optimizations, all samples were tested undiluted in a blind fashion. Luminex 100 (Luminex Corporation, Austin, TX, USA) was used for the immunoassay and Luminex 100 IS software to analyze the bead median fluorescence intensity, as in our recent study (28). All the tests were run in duplicate. 50 µl of each sample or standard solution was added to a 96-well filter plate (provided with the kit) containing 25 µl of antibody-coated fluorescent beads. Biotinylated secondary antibodies and streptavidin-PE were added to the samples with alternate incubation and washing steps. After the last washing, 100 µl of working wash solution was added to the wells. The plate was read on the Luminex 100 array reader. The detection limits for IL-6 and GM-CSF with this method were 29 and 18 pg/ml, respectively. Data were analyzed with the MasterPlex 2.0 software, using a five-PL regression curve to plot the standard curve.

#### RNA Isolation, RT-qPCR

As in previous studies by our group (28, 29), total RNA was prepared using the TRI Reagent (Molecular Research Center, Inc., Cincinnati, OH, USA) and Direct-Zol™ RNA isolation kit (Zymo Research, Irvine, CA, USA) following the manufacturer’s instructions. Then, the samples were treated with DNase I (Zymo Research, Irvine, CA, USA) to remove contaminating genomic DNA and quantified with a NanoDrop ND-2000 spectrophotometer (NanoDrop Technologies, Wilmington, DE, USA). One microgram of total RNA was reverse transcribed with a Maxima™ First Strand cDNA Synthesis Kit for RT-qPCR (Thermo Scientific). Reactions were performed on Stratagene Mx3000P QPCR System (Agilent Technologies, Santa Clara, CA, USA), using glyceraldehyde 3-phosphate dehydrogenase (GAPDH) as the reference gene. GAPDH was chosen because of its stable expression and because it was successfully used in earlier studies to measure relative COX-2 mRNA expression in LPS-induced fever (30–32). Each reaction contained 2 µl of cDNA, 10 µl Luminaris Color HiGreen Low ROX qPCR Master Mix (Thermo Scientific), 0.3 µM of each primer (10 µM), and 6.8 µl of water. The following primer pairs were used to amplify the target loci: GAPDH sense: 5'-TTCACCACCATGGAGAAG-3' and antisense: 5'-GGCATGGACTGTGGTCATGA-3'. COX-2 sense: 5'-GGGTTGCTGGGGGAAGAAA-3' and antisense: 5'- CTCTGCTCTGGTCAATGGAGG-3'. Amplification was carried out under the following conditions: 95°C for 10 min, followed by 40 cycles of 95°C for 30 s, 60°C for 45 s, and 72°C for 45 s. All RT-PCR reactions were carried out in triplicate and included a melt curve analysis to ensure specificity of signal. Relative expression ratios were calculated using MxPro QPCR Software (Agilent Technologies) with the ΔΔCt method, using samples of untreated animals, as a control. Primer efficiencies were taken into account when calculating gene expression ratios (33).

#### Western Blot

Western blot procedures were carried out according to the protocols used in our recent study (34). Lung, liver, and brain tissue samples (~50 mg) were homogenized in ice-cold Tris buffer (50 mM, pH 8.0), containing protease inhibitor cocktail (1:100) and 50 mM of sodium vanadate (Sigma-Aldrich).

Brain samples required additional preparation procedures. To each brain sample, 625 µl of a methanol:chloroform (2:1) mixture was added, and the samples were incubated for 10 min at room temperature with slight agitation. Then, without inhibitors an equal volume (208 µl) of chloroform and Tris buffer (50 mM) was added to each brain sample, and the samples were centrifuged for 10 min (13,000 rpm, room temperature). The supernatants were removed from the protein disks into a new Eppendorf tube, and the bottom layer (containing chloroform) was discarded. Then, the supernatants and protein disks were sonicated on ice twice for 3 s. Trichloroacetic acid was added to the samples at a final concentration of 5% followed by incubation on ice for 10 min. The precipitates were pelleted by centrifugation for 10 min (13,000 rpm, room temperature). The supernatants were discarded, and the pellets were redissolved in Tris buffer (70 mM, pH 8.0). The sonication was repeated as described earlier.

Then, the lung, liver, and prepared brain samples were processed similarly. To each sample, 2× concentrated sodium dodecyl sulfate (SDS)-polyacrylamide gel electrophoresis sample buffer was added. Proteins were separated on 10% SDS-polyacrylamide gel and transferred to nitrocellulose membranes. After blocking (1 h with 2% non-fat milk in Tris-buffered saline), membranes were probed overnight at 4°C with primary antibodies (1:1,000) binding to the 75-kDa COX-2 protein (catalog nr: ab15191; Abcam Plc., Cambridge, UK). Based on the previous studies measuring LPS-induced COX-2 protein expression with Western blot (30, 35, 36), we used GAPDH as the loading reference. Membranes were washed six times for 5 min in Tris-buffered saline, containing 0.1% Tween before addition of goat anti-rabbit horseradish peroxidase-conjugated secondary antibody (1:3,000 dilution; Bio-Rad, Budapest, Hungary). Membranes were washed again as before, and the antibody–antigen complexes were visualized by enhanced chemiluminescence. After scanning, the results were quantified by using ImageJ software (NIH, Bethesda, MD, USA).

### Data Processing and Analysis

Data on deep *T*_b_, tail *T*_sk_, and VO_2_ were compared by three-way ANOVA, while for comparison of serum cytokine levels, COX-2 expression, and PGE_2_ concentrations two-way ANOVA was used. As in our previous studies (25, 26), ANOVA was followed by the Fisher least significant difference *post hoc* test. For statistical analysis, Sigmaplot 11.0 (Systat Software, San Jose, CA, USA) software was used. The effects were considered significant when *P* < 0.05. All data are reported as mean ± SE.

## Results

### Thermoregulatory Phenotype of *Tacr1^+/+^* and *Tacr1^−/−^* Mice

The basal deep *T*_b_ of the mice was nearly identical regardless of either gender or genotype in the thermocouple setup throughout the time period when substance administrations were performed in the fever experiments (Figure S1 in Supplementary Material).

To evaluate whether *Tacr1^−/−^* mice can appropriately activate warmth- and cold-defense mechanisms, we studied the thermoregulatory response of these mice to ambient heating and cooling, respectively. To reveal even a small deficiency in heat defenses, we used a severe heat exposure model that results in ~6°C rise in deep *T*_b_. When exposed to heat, the mice of both genotypes (*Tacr1^+/+^* and *Tacr1^−/−^*) responded with rapid, near-maximal tail skin vasodilation with *T*_sk_ approaching 41°C (Figure S2A in Supplementary Material). Neither the *T*_sk_ response nor the *T*_b_ response differed between the genotypes. Hence, *Tacr1^−/−^* mice are fully capable of increasing heat loss through their tails and defending their deep *T*_b_ against heat.

To reveal even a small deficiency in cold defenses, we used a severe cold exposure model that results in a pronounced drop in deep *T*_b_. When exposed to cold in this model, the mice of both genotypes responded with tail skin vasoconstriction (a decrease in *T*_sk_), but even so their *T*_b_ decreased by ~6°C (Figure S2B in Supplementary Material). The response dynamics did not differ between the two genotypes. Hence, the thermoregulatory response of *Tacr1^−/−^* mice to cooling is unaltered.

### Characteristics of the Thermoregulatory Response of *Tacr1^+/+^* and *Tacr1^−/−^* Mice to LPS

**Figure 1:**
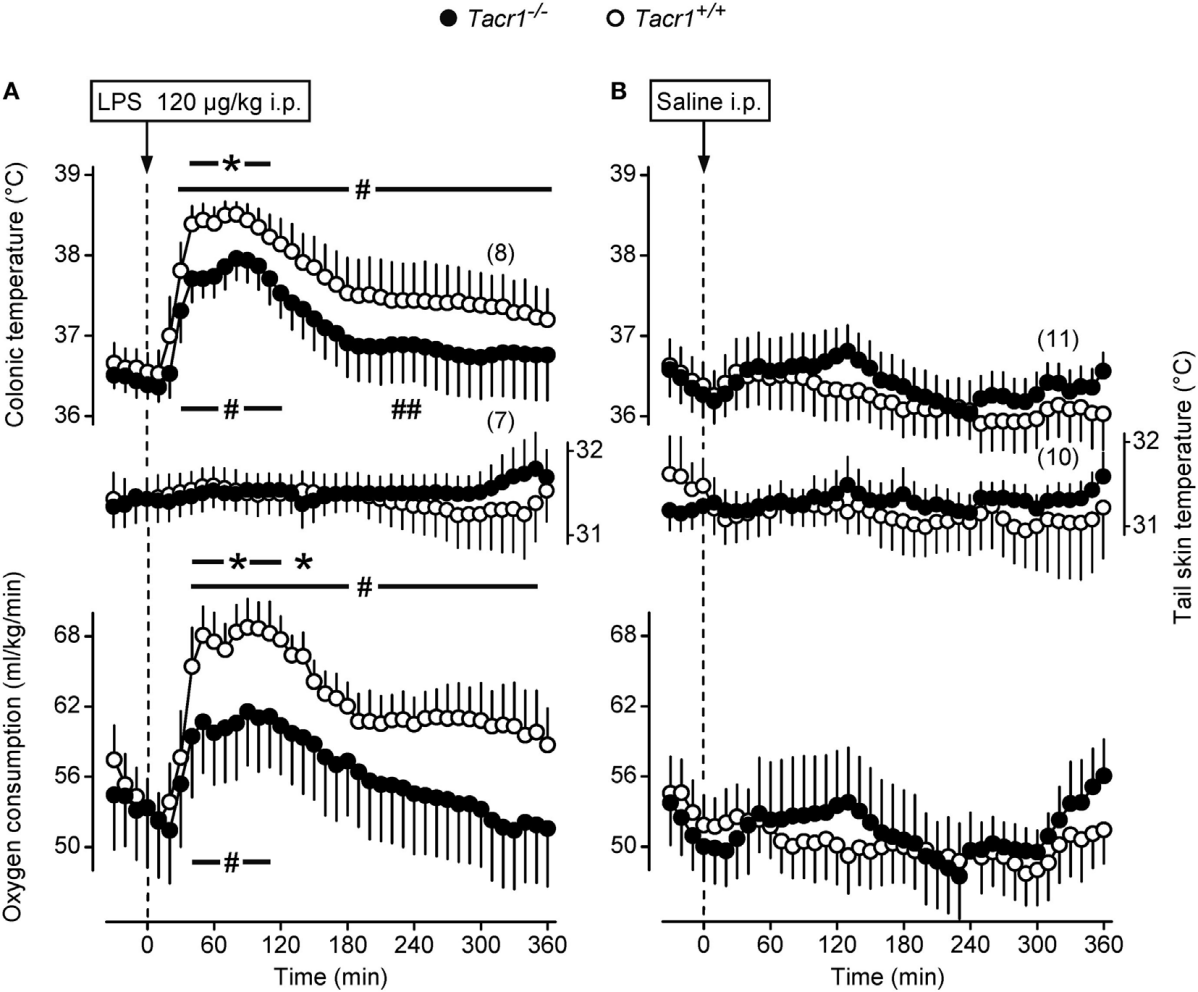
Thermoeffector and colonic temperature responses of *Tacr1^+/+^* and *Tacr1^−/−^* mice to lipopolysaccharide (LPS) **(A)** or saline **(B)** administered intraperitoneally (i.p.). The changes in colonic temperature [a form of deep body temperature (*T*_b_)] are shown in the upper panel; alterations in the activity of the two main autonomic thermoeffectors, skin temperature (*T*_sk_) and rate of oxygen consumption (VO_2_) are depicted in the middle and lower panels, respectively. These experiments were performed in the respirometry setup at an ambient temperature of 31°C. Number of animals in the corresponding groups are indicated in the figure. *P < 0.05, intergenotype difference in the response to LPS. ^#^*P* < 0.05, LPS vs. saline difference within the same genotype as determined by the Fisher LSD test.

In thermophysiological experiments, we compared the fever response between *Tacr1^+/+^* and *Tacr1^−/−^* mice. When treated with LPS (120 µg/kg, i.p.), the mice of both genotypes developed fever as compared to saline-treated mice (Figures 1A,B). However, the effects of both the treatment × time interaction [ANOVA, *F*_(42,1376)_ = 2.114, *P* < 0.001] and treatment × genotype interaction [*F*_(1,1376)_ = 40.908, *P* < 0.001] were significant on their *T*_b_ response. LPS-treated *Tacr1^+/+^* mice responded with a typical fever response: their deep *T*_b_ started to increase at 20 min, plateaued (~38.5°C) between 40 and 100 min, then it gradually decreased, but remained elevated compared to saline treatment throughout the experiment (Fisher LSD test, *P* < 0.05 at 30–360 min). These findings are in line with those reported previously (37). In *Tacr1^−/−^* mice, the LPS-induced fever response was less pronounced than in *Tacr1^+/+^* mice, reaching the level of significance at 40–110 min compared to saline treatment (Fisher LSD test, *P* < 0.05) (Figure 1A). The LPS-induced increase in deep *T*_b_ was brought about by an elevation of VO_2_, changing with parallel dynamics as *T*_b_ in both genotypes (Figures 1A,B). Similar to *T*_b_, the effects of both the treatment × time interaction [ANOVA, *F*_(42,1161)_ = 1.618, *P* < 0.01] and treatment × genotype interaction [*F*_(1,1161)_ = 15.802, *P* < 0.001] were significant on the VO_2_ of the mice. After LPS treatment, the VO_2_ was significantly higher compared to saline at 40–350 min in *Tacr1^+/+^* mice (Fisher LSD test, *P* < 0.05) and at 40–140 min in *Tacr1^−/−^* mice (Fisher LSD test, *P* < 0.05). Since the experiments were carried out at the lower end of the thermoneutral zone, the mice exhibited cutaneous vasoconstriction (as indicated by their low tail *T*_sk_), thus no further decrease in tail *T*_sk_ was observed. Importantly, the *T*_b_ of the LPS-treated *Tacr1^−/−^* mice was markedly (0.5–0.7°C) lower than that of *Tacr1^+/+^* mice starting from 40 min post-LPS infusion until the end of the experiment. Parallel with *T*_b_, the LPS-induced elevation of VO_2_ was also suppressed in *Tacr1^−/−^* mice compared to their *Tacr1^+/+^* littermates (Figure 1A). The LPS-induced elevation of both parameters was significantly attenuated in *Tacr1^−/−^* mice compared to *Tacr1^+/+^* mice at 40–120 min (Fisher LSD test, *P* < 0.05 for intergenotype difference) (Figure 1A). The infusion of saline did not cause any effect on deep *T*_b_, tail *T*_sk_, and VO_2_ in the mice of either genotype (Figure 1B). Our results demonstrate that LPS-induced fever is attenuated in *Tacr1^−/−^* mice already in the early stage (i.e., starting from ~40 min). Next, we wanted to know the suppression of which part of the “pyrogenic cytokine-COX-2-PGE_2_ axis” is responsible for attenuating the fever response in the absence of *Tacr1*.

### The Thermoregulatory Effects of PGE_2_ and SP in *Tacr1^+/+^* and *Tacr1^−/−^* Mice

**Figure 2:**
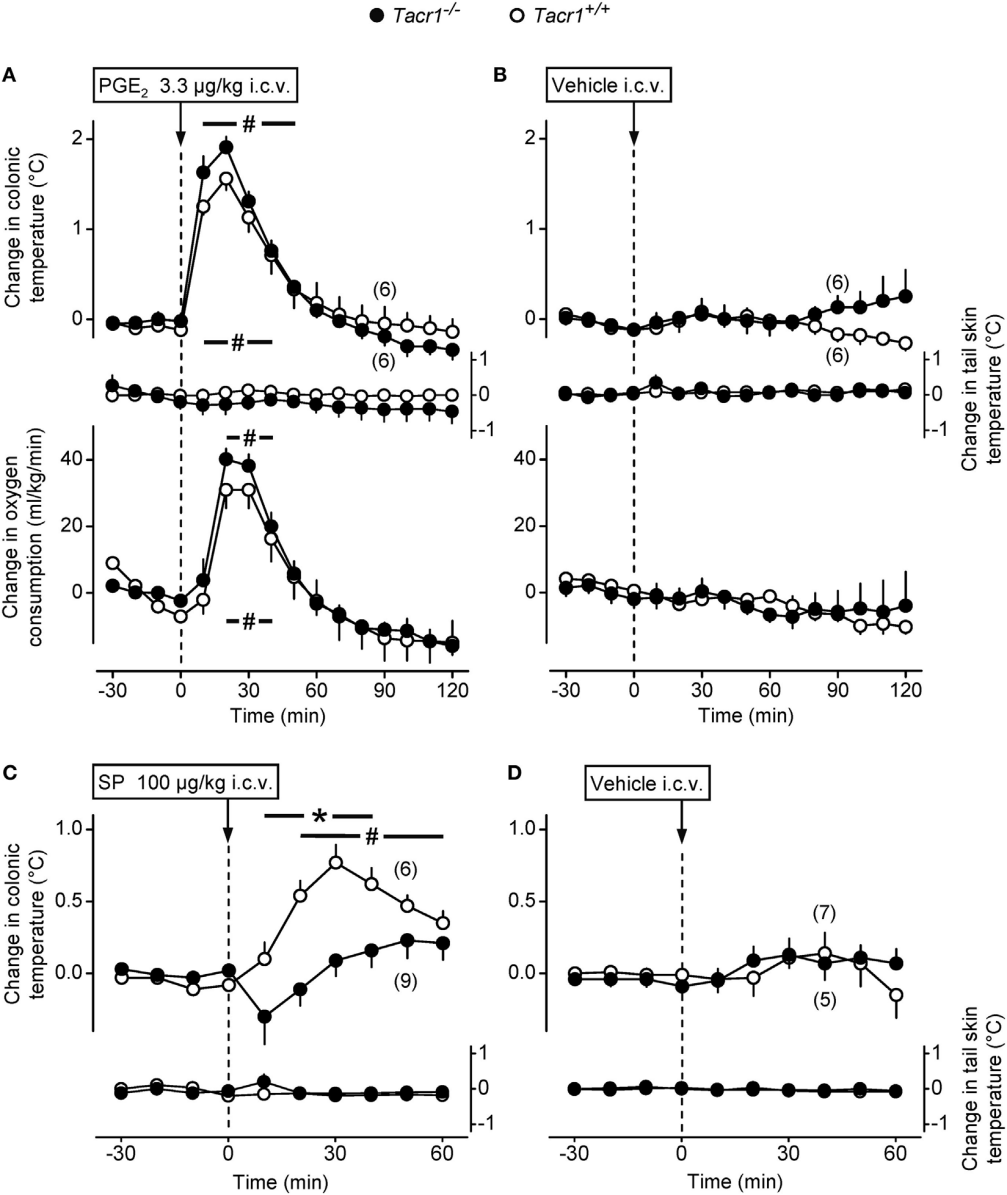
The thermoregulatory response of *Tacr1^+/+^* and *Tacr1^−/−^* mice to prostaglandin (PG) E_2_ and substance P (SP) administered intracerebroventricularly (i.c.v.). Changes in colonic temperature (upper panel), skin temperature (*T*_sk_) (middle panel), and rate of oxygen consumption (VO_2_) (bottom panel) in response to PGE_2_ (dose indicated) **(A)** and its vehicle **(B)** in *Tacr1^+/+^* and *Tacr1^−/−^* mice. The experimental conditions were identical to those described in Figure 1 (respirometry setup, ambient temperature of 31°C). At the time of the injection, the values of colonic temperature of the *Tacr1^+/+^* and *Tacr1^−/−^* mice were, respectively, 37.0 ± 0.2 and 37.1 ± 0.1°C for PGE_2_-treated mice and 36.8 ± 0.3 and 36.9 ± 0.2°C for vehicle-treated mice. These values did not differ statistically from each other. Changes in colonic temperature and *T*_sk_ in response to SP (dose indicated) **(C)** and its vehicle **(D)** in *Tacr1^+/+^* and *Tacr1^−/−^* mice. These experiments were performed in the thermocouple setup at an ambient temperature of 33°C. At the time of the injection, the values of colonic temperature of the *Tacr1^+/+^* and *Tacr1^−/−^* mice were, respectively, 37.2 ± 0.2 and 37.1 ± 0.2°C for SP-treated mice and 37.3 ± 0.3 and 37.3 ± 0.1°C for vehicle-treated mice. These values did not differ statistically from each other. Number of animals in the corresponding groups are indicated in the figure. *P < 0.05, intergenotype difference in the response to SP. ^#^*P* < 0.05, treatment (PGE_2_ or SP) vs. vehicle difference within the same genotype as determined by the Fisher LSD test.

First, we studied whether the effect of PGE_2_, i.e., the key mediator of fever (2), is also reduced in the *Tacr1^−/−^* mice. As the main site of the febrigenic action of PGE_2_ is situated in the preoptic area of the hypothalamus (10), we compared the thermoregulatory effects of i.c.v. injected PGE_2_ between *Tacr1^+/+^* and *Tacr1^−/−^* mice. In response to PGE_2_ the mice of both genotypes rapidly developed a marked elevation in deep *T*_b_ and VO_2_ (Figure 2A), while administration of the vehicle did not cause any effects in either genotype (Figure 2B). The effect of the treatment × time interaction was significant with regard to both *T*_b_ and VO_2_ [ANOVA, *F*_(18,380)_ = 29.406, *P* < 0.001 and *F*_(18,380)_ = 11.922, *P* < 0.001, respectively], whereas the effects of the genotype or the treatment × genotype interaction were not significant on either deep *T*_b_ or VO_2_. Of note, there was no attenuation in either the *T*_b_ or VO_2_ rise in the *Tacr1^−/−^* mice compared to their *Tacr1^+/+^* littermates. These experiments were conducted under identical conditions as those with LPS, including the ambient temperature (31°C) near the lower end of the thermoneutral zone, which can explain why we did not observe any change in the tail *T*_sk_ in the mice of either genotype. Our results with PGE_2_ rule out the possibility that *Tacr1^−/−^* mice are unable to activate their thermogenesis and increase their *T*_b_ to stimuli other than LPS. More importantly, the lack of attenuation in the PGE_2_-induced thermoregulatory response of the *Tacr1^−/−^* mice suggests that their reduced fever response to LPS is due to a more upstream suppression in the febrigenic molecular pathway.

In a separate set of experiments, we confirmed the absence or presence of functional NK1 receptors in the *Tacr1^−/−^* and *Tacr1^+/+^* mice, respectively. It has been repeatedly shown that SP evokes an increase in deep *T*_b_ by acting on the NK1 receptor (15, 16), therefore, we injected SP (100 µg/kg, i.c.v.) or its vehicle to *Tacr1^+/+^* and *Tacr1^−/−^* mice and compared their *T*_b_ responses (Figures 2C,D). We found that the SP-induced increase in deep *T*_b_ was practically absent in *Tacr1^−/−^* mice, whereas it was significant in *Tacr1^+/+^* mice at 20–60 min compared to vehicle treatment (Fisher LSD test, *P* < 0.05) (Figures 2C,D). The effects of both the genotype [ANOVA, *F*_(1,260)_ = 10.538, *P* < 0.01] and the treatment × genotype interaction [*F*_(1,260)_ = 13.233, *P* < 0.001] were significant. In the SP-treated mice, there was a significant difference between the *T*_b_ of *Tacr1^+/+^* and *Tacr1^−/−^* mice at 10–40 min (Fisher LSD test, *P* < 0.05), which confirms the validity of this animal model.

### LPS-Induced Changes in Serum Cytokine Levels of *Tacr1^+/+^* and *Tacr1^−/−^* Mice

**Figure 3:**
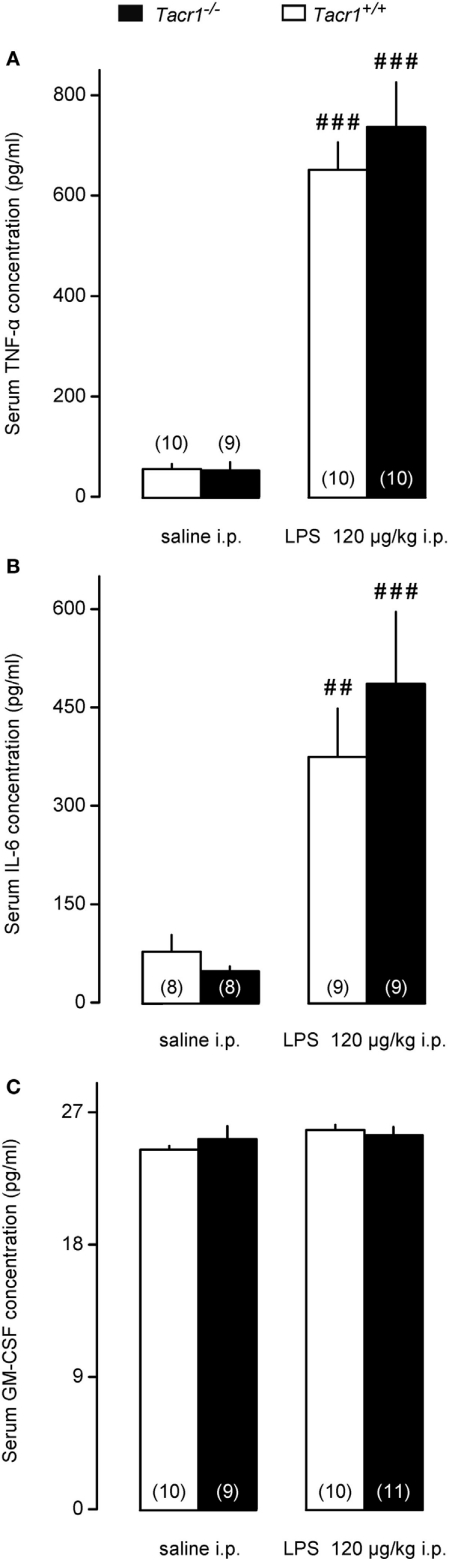
Serum cytokine concentrations in *Tacr1^+/+^* and *Tacr1^−/−^* mice. **(A)** Serum tumor necrosis factor (TNF)-α concentrations in *Tacr1^+/+^* and *Tacr1^−/−^* mice in response to lipopolysaccharide (LPS) (dose indicated) or saline. **(B)** Serum IL-6 concentrations in *Tacr1^+/+^* and *Tacr1^−/−^* mice in response to LPS (dose indicated) or saline. **(C)** Serum granulocyte-macrophage colony-stimulating factor (GM-CSF) concentrations in *Tacr1^+/+^* and *Tacr1^−/−^* mice in response to LPS (dose indicated) or saline. Blood samples were collected at 40 min postinfusion. Number of animals in the corresponding groups are indicated in the figure. Within each genotype, significant differences in the response to LPS (as compared to saline) are marked as ^##^*P* < 0.01 or ^###^*P* < 0.001 as determined by the Fisher LSD test.

One of the early steps in LPS-induced fever signaling is the activation of innate immune cells, including macrophages, leukocytes, and dendritic cells, which, in turn leads to augmented production of inflammatory cytokines, including TNF-α and IL-6 (3). Thus, in our next experiments we measured serum concentrations of TNF-α and IL-6 in the *Tacr1^+/+^* and *Tacr1^−/−^* mice to assess whether the LPS-induced cytokine production is suppressed in the absence of the NK1 receptor. The serum concentrations of TNF-α and IL-6 did not differ between the genotypes after the infusion of saline (Figures 3A,B). The administration of LPS resulted in a substantial upsurge of TNF-α (Fisher LSD test, *P* < 0.001 vs. saline for both genotypes) (Figure 3A) and IL-6 (*P* < 0.01 for *Tacr1^+/+^* mice and *P* < 0.001 for *Tacr1^−/−^* mice) (Figure 3B). Importantly, we did not detect any significant difference in the TNF-α and IL-6 concentrations between *Tacr1^+/+^* and *Tacr1^−/−^* mice (Figures 3A,B).

It was shown that LPS can induce the secretion of GM-CSF in mice, which, in turn, can stimulate the production of inflammatory cytokines, such as TNF-α (38). We wanted to know whether changes in serum GM-CSF concentration can play a role in the attenuated fever response of the *Tacr1^−/−^* mice to LPS. In our experimental model, we did not find any difference in the serum concentrations of GM-CSF between LPS-treated and saline-treated mice of either genotype (Figure 3C). The absence of a surge in GM-CSF concentration in response to LPS could be due to the early time point (40 min post-LPS infusion) chosen for blood collection in our experiments.

These data indicate that impaired pyrogenic cytokine production does not contribute to the attenuated fever response of *Tacr1^−/−^* mice to LPS.

### LPS-Induced Changes in COX-2 Expression in *Tacr1^+/+^* and *Tacr1^−/−^* Mice

#### Changes in COX-2 mRNA Expression in the Lungs, Liver, and Brain of *Tacr1^+/+^* and *Tacr1^−/−^* Mice

**Figure 4:**
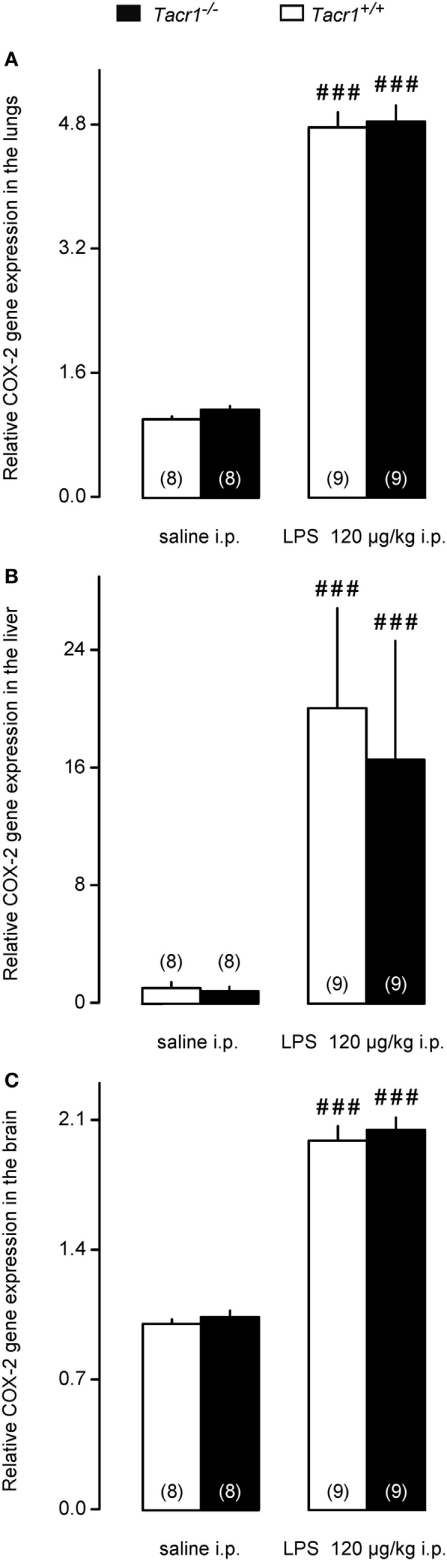
Relative cyclooxygenase-2 (COX-2) gene expression in the lungs **(A)**, liver **(B)**, and in the brain **(C)** of *Tacr1^+/+^* and *Tacr1^−/−^* mice after infusion of lipopolysaccharide (LPS) (dose indicated) or saline. Tissue samples were collected at 40 min postinfusion. Number of animals in the corresponding groups are indicated in the figure. Within each genotype, significant differences in the response to LPS (as compared to saline) are marked as ^###^*P* < 0.001 as determined by the Fisher LSD test.

We moved downstream in the fever signaling pathway and compared the LPS-induced expression of COX-2 between *Tacr1^+/+^* and *Tacr1^−/−^* mice. At the mRNA level, COX-2 expression is upregulated in rats already in the early phase of the febrile response (~30–40 min after LPS infusion) both in peripheral organs (lungs and liver) and to a lesser extent in the brain (31). In the present study, our experiments with LPS revealed that the fever response of *Tacr1^−/−^* mice was attenuated already at ~40 min post-LPS infusion (Figure 1A), and therefore, we collected lung, liver, and brain samples at this time point and studied the COX-2 mRNA expression in these tissues. As compared with the values of expression in saline-treated mice, the administration of LPS caused transcriptional upregulation of COX-2 mRNA in the lungs (Figure 4A), in the liver (Figure 4B), and in the brain (Figure 4C) of *Tacr1^+/+^* and *Tacr1^−/−^* mice (Fisher LSD test, *P* < 0.001 vs. saline for all three tissues). There was no significant difference between the genotypes in any of the three tissue samples. The magnitude of the LPS-induced increase in the level of COX-2 expression was not the same in the three organs studied: ~5-fold in the lungs, 17–20-fold in the liver, and 2-fold in the brain (Figures 4A–C). These results are well in accordance with earlier findings on the dynamics of LPS-induced COX-2 mRNA expression (31), but cannot explain the attenuation of the febrile response in the *Tacr1^−/−^* mice.

#### Changes in COX-2 Protein Expression in the Lungs, Liver, and Brain of *Tacr1^+/+^* and *Tacr1^−/−^* Mice

**Figure 5:**
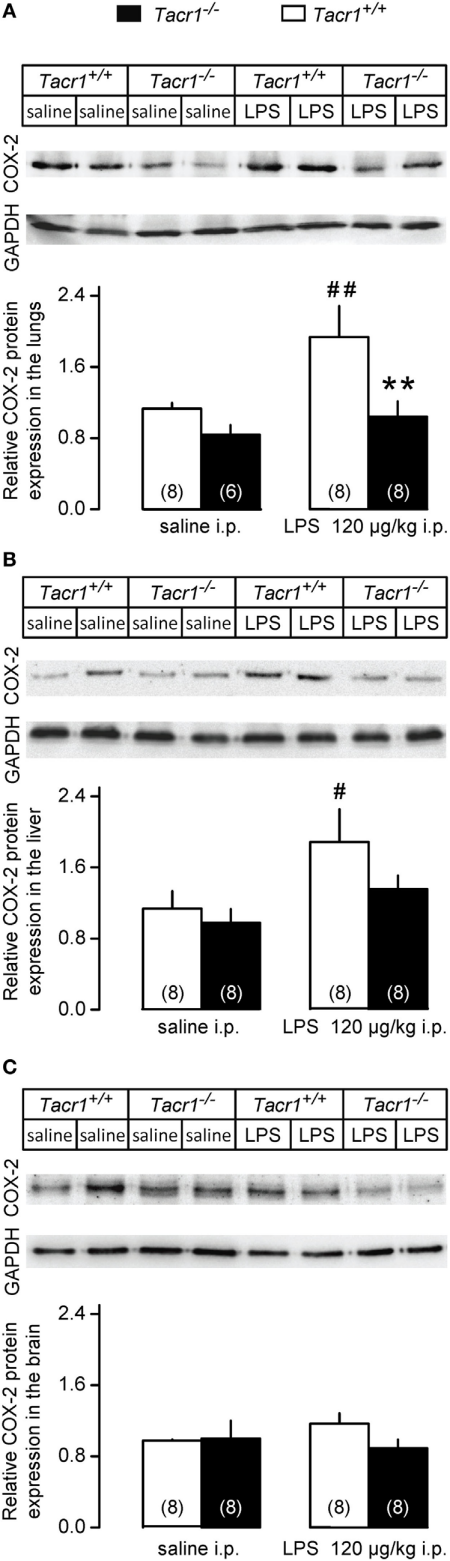
Relative cyclooxygenase-2 (COX-2) protein expression in the lungs **(A)**, liver **(B)**, and in the brain **(C)** of *Tacr1^+/+^* and *Tacr1^−/−^* mice after infusion of lipopolysaccharide (LPS) (dose indicated) or saline. Tissue samples were collected at 40 min postinfusion. Number of animals in the corresponding groups are indicated in the figure. **P < 0.01, intergenotype difference in the response to LPS; ^#^*P* < 0.05 or ^##^*P* < 0.01, LPS vs. saline difference within the same genotype as determined by the Fisher LSD test.

Besides the LPS-induced amplified expression of COX-2 mRNA, the expression of the COX-2 protein was also found to increase in the periphery (but not in the brain) already in the early phase of the febrile response (9). Therefore, we also compared the COX-2 protein expression between LPS-treated *Tacr1^+/+^* and *Tacr1^−/−^* mice (Figure 5). In the lungs, the effects of both the treatment [ANOVA, *F*_(1,26)_ = 6.165, *P* < 0.05] and genotype [*F*_(1,26)_ = 8.532, *P* < 0.01] were significant (Figure 5A). In the liver, the effect of treatment was also significant [ANOVA, *F*_(1,28)_ = 6.555, *P* < 0.05] (Figure 5B), whereas no significant change was found in the brain (Figure 5C). In *Tacr1^+/+^* mice, the administration of LPS resulted in a marked increase in COX-2 protein expression in the lungs (Fisher LSD test, *P* < 0.01 vs. saline) and in the liver (Fisher LSD test, *P* < 0.05), but not in the brain (Fisher LSD test, *P* = 0.264). In contrast with the *Tacr1^+/+^* mice, the COX-2 protein expression did not change significantly in either the lungs or the liver of LPS-treated *Tacr1^−/−^* mice as compared to saline treatment. In LPS-treated mice, the COX-2 protein expression was attenuated in the lungs of *Tacr1^−/−^* mice compared with their *Tacr1^+/+^* littermates (Fisher LSD test, *P* < 0.01), while in the liver there was a tendency for reduced COX-2 expression in the LPS-treated *Tacr1^−/−^* mice (Fisher LSD test, *P* = 0.101). These findings indicate that the absence of the NK1 receptor interferes with the augmentation of COX-2 expression at the protein level.

### Changes in PGE_2_ Concentration in the Lungs, Liver, and Brain of *Tacr1^+/+^* and *Tacr1^−/−^* Mice

**Figure 6:**
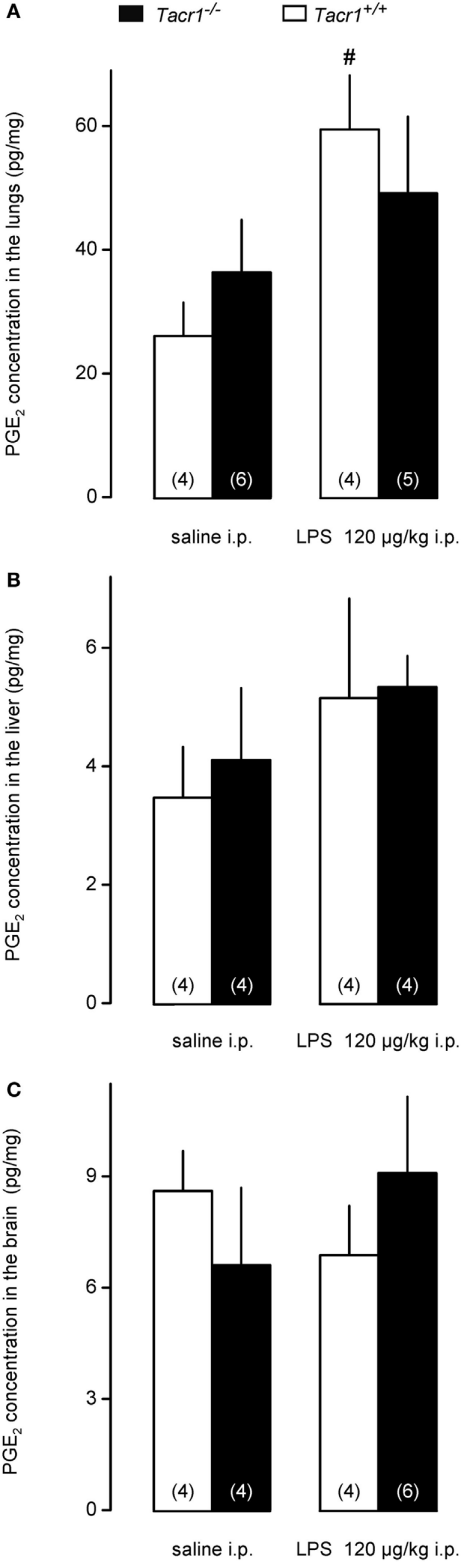
Prostaglandin (PG) E_2_ concentration in the lungs **(A)**, liver **(B)**, and in the brain **(C)** of *Tacr1^+/+^* and *Tacr1^−/−^* mice after infusion of lipopolysaccharide (LPS) (dose indicated) or saline. Tissue samples were collected at 40 min postinfusion. Number of animals in the corresponding groups are indicated in the figure. ^#^*P* < 0.05, LPS vs. saline difference within the same genotype as determined by the Fisher LSD test.

To assess whether the attenuated expression of COX-2 protein results in reduced production of PGE_2_ in the LPS-treated *Tacr1^−/−^* mice, we measured PGE_2_ concentrations in the lungs, liver, and brain of the mice. We found that the LPS treatment resulted in the biggest (~80%) increase in PGE_2_ concentration in the lungs (Figure 6A), followed by the liver (~40%) (Figure 6B), and then by the brain (~10%) (Figure 6C). The effect of treatment was significant in the lungs [ANOVA, *F*_(1,15)_ = 7.065, *P* < 0.05], but not in the other two organs. *Post hoc* analysis revealed that the LPS-induced increase in PGE_2_ concentration was significant in the lungs of *Tacr1^+/+^* mice compared with saline (Fisher LSD test, *P* < 0.05), whereas in the lungs of *Tacr1^−/−^* mice the PGE_2_ level did not increase significantly in response to LPS (Fisher LSD test, *P* = 0.275 vs. saline). These results suggest that in the lungs of *Tacr1^−/−^* mice the LPS-induced surge in PGE_2_ concentration is reduced.

## Discussion

In our study, we showed that the absence of the NK1 receptor results in the attenuation of LPS-induced fever for the first time by using *Tacr1^−/−^* mice. Our experimental model allowed us to detect the suppression of the febrile response already in the early phase of fever (starting from ~40 min post-LPS infusion), which is also a novel finding. When we looked at the molecular mechanism, we did not find a difference in the PGE_2_-induced febrile response between *Tacr1^+/+^* and *Tacr1^−/−^* mice. The LPS-induced serum cytokine production and COX-2 mRNA expression in the lungs, liver, and brain of the mice were also statistically indistinguishable between the genotypes. In contrast with mRNA, when we measured COX-2 expression at the protein level, we found that the LPS-induced surge was significantly attenuated in the lungs and tended to be suppressed in the liver of *Tacr1^−/−^* mice as compared with their *Tacr1^+/+^* littermates.

The involvement of SP signaling and the NK1 receptor in experimental fever was reported in earlier studies (13–16, 39). Antagonists of SP reduced the febrile response to LPS in rats and guinea pigs from the beginning of the response, which was detectable 45–90 min after LPS infusion (13, 14). However, when the authors looked at the mechanisms connecting the NK1 receptor with the fever signaling pathway, they focused on the later phases of fever (i.e., 2 h or more post-LPS infusion), presumably, because the early phase was absent in their experiments due to stress–hyperthermia as a consequence of stressful (needle-pinch) drug injection (16). In the present study, we conducted the experiments under such conditions (extensive habituation, moderate LPS dose, non-stressful substance administration, and near neutral ambient temperature) that allowed us to study LPS-induced fever from 40 to 360 min postinfusion in mice, thus we could detect the attenuation of the response already at 40 min in the absence of *Tacr1* gene. A caveat in knockout mouse models is that compensatory mechanisms may develop. With regard to alteration of other NK receptors in mice genetically lacking the NK1 receptor, it was shown with RT-PCR and immunostaining that the expression of NK3 receptors was unchanged in the retina of *Tacr1^−/−^* mice as compared with *Tacr1^+/+^* mice (40). However, in a mouse model of meningoencephalitis, the effects of combined treatment with NK2 and NK3 receptor antagonists were reduced on the neuroinflammatory scores in *Tacr1^−/−^* mice compared with similarly treated *Tacr1^+/+^* mice (41). It was concluded that in the genetic absence of NK1 receptors, tachykinins may utilize NK2 and NK3 receptors (41), although expression of the NK receptors was not measured. Taken together, in *Tacr1^−/−^* mice the expression of other NK receptors is presumably unchanged, but there might be an alteration in the utilization of NK2 and NK3 receptor-mediated mechanisms, which warrants for further characterization of the tachykinin pathways in this mouse model.

The later phases of fever are mediated mostly by increased PGE_2_ production in the brain, and it is well established that brain-derived PGE_2_ is an important mediator for the maintenance of LPS-induced fever (32, 42–44). However, the early phase of fever is triggered from peripheral organs such as the lungs and the liver (9, 31). Therefore, our results suggest that the genetic blockade of the NK1 receptor interferes with fever signaling at a peripheral site of action in the early phase of LPS-induced fever. We further supported the peripheral action site of NK1 receptor in fever by showing that the *Tacr1^−/−^* mice were equally able to increase their thermogenesis and deep *T*_b_ in response to i.c.v. administration of PGE_2_. We focused the second part of our study on exploring which step of fever signaling is altered in the *Tacr1^−/−^* mice. In the periphery, SP signaling has been shown to play a role in the induction of pyrogenic cytokine production by macrophages (45) and in pulmonary macrophage activation (46). In acute lung injury after burns, SP was found to upregulate COX-2 activity (47). The expression of the NK1 receptor by macrophages is well documented (48–53), but it was also found in various other immune cells (1). With regard to leukocytes, neutrophil accumulation was significantly inhibited in *Tacr1^−/−^* mice in lung injury induced by immune complexes (54) or acute pancreatitis (55). Attenuated leukocyte recruitment and lung injury were also observed with the NK1 receptor antagonist SR140333 in a murine model of polymicrobial sepsis induced by cecal ligation and puncture (56). In the same model, the authors later showed that the SP-induced pro-inflammatory response was mediated mainly by protein kinase C (PKC)-α (57). Whether the NK1 receptor-mediated leukocyte recruitment occurs *via* a direct action on granulocytes or indirectly through other cell types (e.g., endothelial cells and bronchial epithelial cells) remains a question of debate [for a review, see Ref. (58)]. On the one hand, the expression of functional NK1 receptors was demonstrated on granulocytes in mice (59) and humans (60). In the latter study, it was also shown that the SP-induced COX-2 expression was mediated by NK1 receptors (60). On the other hand, different authors failed to detect the presence of NK1 receptors in human granulocytes (52, 61), which indicates that the NK1 receptor-mediated granulocyte migration develops *via* a primary effect of SP on other cell types. In line with such scenario, expression of the NK1 receptor was shown in several types of stromal cells in the lung, including bronchial glands, bronchial vessels, and bronchial smooth muscle (62), as well as airway epithelial cells (63, 64) and postcapillary venular endothelial cells (65). With the help of nested PCR, the expression of the NK1 receptor was demonstrated also in the liver, predominantly in non-parenchymal cells, most likely macrophages, lymphocytes, and granulocytes, but also in hepatocytes (53), which contradicted earlier studies reporting no detectable expression of the NK1 receptor in the liver by using classical techniques (66–68). In lung epithelial cell cultures, SP *via* NK1 receptors stimulated neutrophil adherence (69) and pro-inflammatory cytokine production (70). Moreover, LPS enhanced the SP-induced neutrophil adherence and associated cytokine release *via* an involvement of NK1 receptors (71). Taken together, it is possible that LPS-induced leukocyte trafficking is reduced in *Tacr1^−/−^* mice, which may be caused either by a direct effect of the NK1 receptor’s absence on leukocytes or indirectly through other cell types such as lung epithelial cells. The reduced trafficking and therefore a reduced cellular infiltrate within the lung and the liver might contribute to reduced expression of COX-2.

As discussed earlier, many peripheral events of the fever response can be influenced by SP signaling. In the present study, we did not find difference in the serum levels of inflammatory cytokines (TNF-α and IL-6) between the LPS-treated *Tacr1^+/+^* and *Tacr1^−/−^* mice, which indicates that the activation of macrophages and their cytokine production is not impaired in the absence of the NK1 receptor. We did not measure the levels of the third major pro-inflammatory cytokine, IL-1β, because it has been shown that it exerts its pyrogenic actions independently from the NK1 receptor (16). LPS can also modulate COX-2 transcriptionally and posttranscriptionally in macrophages independently from inflammatory cytokines (72). When we determined the COX-2 mRNA expression, we found that at this early time point (~40 min) it was greatly amplified in the lungs and liver, and to lesser extent in the brain of the mice, which results are in harmony with the previous findings (31). The lack of difference between the genotypes indicates that transcriptional upregulation of COX-2 is not influenced by the NK1 receptor. The correlation of mRNA and protein levels in biological samples is often poor (73), moreover, the expression of COX-2 is regulated not only at the level of transcription but also at the levels of post-transcription and translation (72, 74, 75). Therefore, we also determined the expression of the COX-2 protein in the lungs, liver, and brain of the mice. In accordance with previous reports showing augmented expression of the COX-2 protein in the periphery (9), we also detected LPS-induced amplification of the COX-2 protein expression in the lungs and liver of *Tacr1^+/+^* mice as compared with saline treatment, whereas we did not find significant increase in their brain. Importantly, however, the LPS-induced amplification of the expression of the COX-2 protein was attenuated in the lungs and tended to be suppressed in the liver of *Tacr1^−/−^* mice as compared with their *Tacr1^+/+^* littermates. In accordance with the different COX-2 protein expression between the genotypes, the administration of LPS caused a significant surge of PGE_2_ concentration in the lungs of *Tacr1^+/+^* mice, which was absent in the *Tacr1^−/−^* mice. It can be expected that the observed difference in pulmonary PGE_2_ synthesis also results in different plasma concentrations of PGE_2_ between the genotypes. The sensitive site where PGEs produce fever is located within the region of the brain that includes the organum vasculosum laminae terminalis and the surrounding preoptic area of the hypothalamus (76). Peripherally borne PGE_2_ can broadly penetrate in the perivascular space in the periventricular organs (such as the organum vasculosum laminae terminalis) and activate neurons or non-neural cells, thus trigger the febrile response (77). It has to be noted that fever signaling was not examined at later time points in the current experiments due to the study design, and therefore it cannot be excluded that COX-2 expression and PGE_2_ production in the brain are also affected by the blockade of the NK1 receptor, especially during the maintenance phase of fever. The experimental confirmation of the LPS-induced temporospatial distribution of PGE_2_ in the plasma, cerebrospinal fluid, and specific brain regions in the absence of the NK1 receptor remains subjects for future studies.

Our results demonstrate for the first time that at the onset of the fever response the NK1 receptor contributes to the augmentation of COX-2 protein expression in peripheral organs. In accordance with our findings, the modulation of COX-2 protein expression by SP signaling has been shown in several human cell lines, including polymorphonuclear leukocytes (60), colonic epithelial cells (78), and endothelial cells (79). Furthermore, an autocrine circuitry between SP and PGE_2_ production was recently suggested in the fever response to endogenous pyrogens (39). The exact mechanism, how SP signaling interacts with COX-2 expression remains to be elucidated in future studies. It is possible that the absence of the NK1 receptor leads to alterations in the expression of enzymes upstream (e.g., PLA_2_) or downstream of COX-2 (e.g., PGE synthases). An alteration in the phosphorylation (i.e., activation) of the cytosolic (c) form of PLA_2_ could be of particular interest, as in the fundamental study by Steiner et al. (9) LPS increased the contents of phosphorylated cPLA_2_ and COX-2 in the lung, but did not alter the protein level of constitutively expressed microsomal PGE_2_ synthase-1. In Chinese hamster ovary cells stably expressing NK1 receptors, SP induced the release of arachidonic acid, presumably by the activation of cPLA_2_, which was blocked by an antagonist of the NK1 receptor (80). Furthermore, in a mouse model of nerve injury, the enhanced activation of cPLA_2_ was abolished by NK1 receptor antagonist in neurons and possibly microglia in the spinal cord (81). These data indicate a link between activation of cPLA_2_ and NK1 receptor-mediated SP signaling at least in some cell types. Further studies are needed to assess whether the LPS-induced phosphorylation of cPLA_2_ is altered in the lungs of *Tacr1^−/−^* mice in addition to the decreased COX-2 protein expression as shown in this study. A potential link in signal transduction between SP and COX-2 may coexist through PKC, as it was shown earlier that inhibitory effects of SP are mediated, at least in part by PKC isoenzymes (82), which play a key role in the biosynthesis of PGE_2_, likely by regulating the induction of COX-2 (83).

The findings of the present study further advance our understanding about the interactions between SP signaling and the “cytokine-COX-2-PGE_2_” axis in experimental fever. As a perspective, our results can help to identify NK1 receptor as a drug target to suppress peripheral COX-2 activity.

## Ethics Statement

All procedures were conducted under protocols approved by Institutional Animal Use and Care Committee of the University of Pecs and were in accordance with the directives of the National Ethical Council for Animal Research and those of the European Communities Council (86/609/EEC).

## Author Contributions

Eszter P, PH, Erika P, and AG designed the study; Eszter P, VT, CZ, ZR, EO, PK, NK, RM, LD, KO, NS, KP, AK, and AG performed experiments; PH and Erika P provided compounds, mice, and analytic tools; Eszter P, VT, EO, RM, LD, KP, AK, and AG processed and analyzed data; and Eszter P, VT, LD, KP, AK, and AG wrote the paper. All the authors reviewed and finally approved the manuscript.

## Conflict of Interest Statement

The authors declare that the research was conducted in the absence of any commercial or financial relationships that could be construed as a potential conflict of interest. The reviewer DC and handling editor declared their shared affiliation.

## Abbreviations

COX, cyclooxygenase; GAPDH, glyceraldehyde 3-phosphate dehydrogenase; GM-CSF, granulocyte-macrophage colony-stimulating factor; i.c.v., intracerebroventricular(ly); i.p., intraperitoneal(ly); IL, interleukin; LPS, lipopolysaccharide; NK, neurokinin; PG, prostaglandin; PKC, protein kinase C; PLA_2_, phospholipase A_2_ (e.g., cytosolic, cPLA_2_); SP, substance P; *T*_b_, body temperature; *T*_sk_, skin temperature; PE, polyethylene; TNF, tumor necrosis factor; VO_2_, rate of oxygen consumption.
