# Supplementary material for: The Neurokinin-1 Receptor Contributes to the Early Phase of Lipopolysaccharide-Induced Fever via Stimulation of Peripheral Cyclooxygenase-2 Protein Expression in Mice
Source: Front Immunol. 2018 Feb 5;9:166. doi: 10.3389/fimmu.2018.00166 (PMC5807668; doi:10.3389/fimmu.2018.00166)
Supplement: Supplementary file 1 [file Image_1.PDF]

Supplementary figures

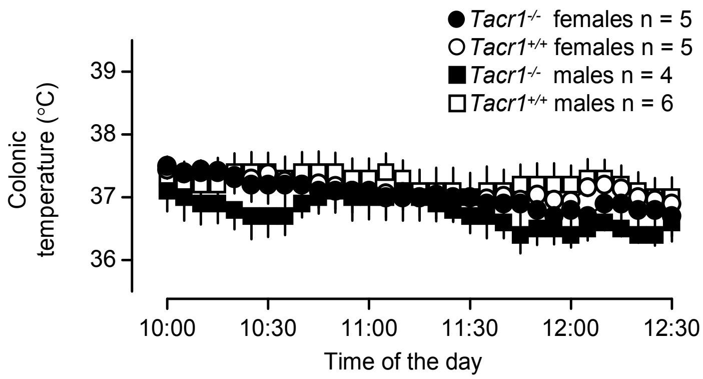

Supplementary figure 1.

Basal colonic temperature of loosely restrained *Tacr1*$^{+/+}$ and *Tacr1*$^{-/-}$ mice of both sexes. Recordings of colonic temperature were performed between 10:00 A.M. and 12:30 P.M (*i.e.*, the time interval of substance administration in the study) in the thermocouple setup at an ambient temperature of 33°C. The colonic temperatures of the mice were identical in all groups of mice throughout the recordings. Numbers of animals in the corresponding groups are indicated in the figure.

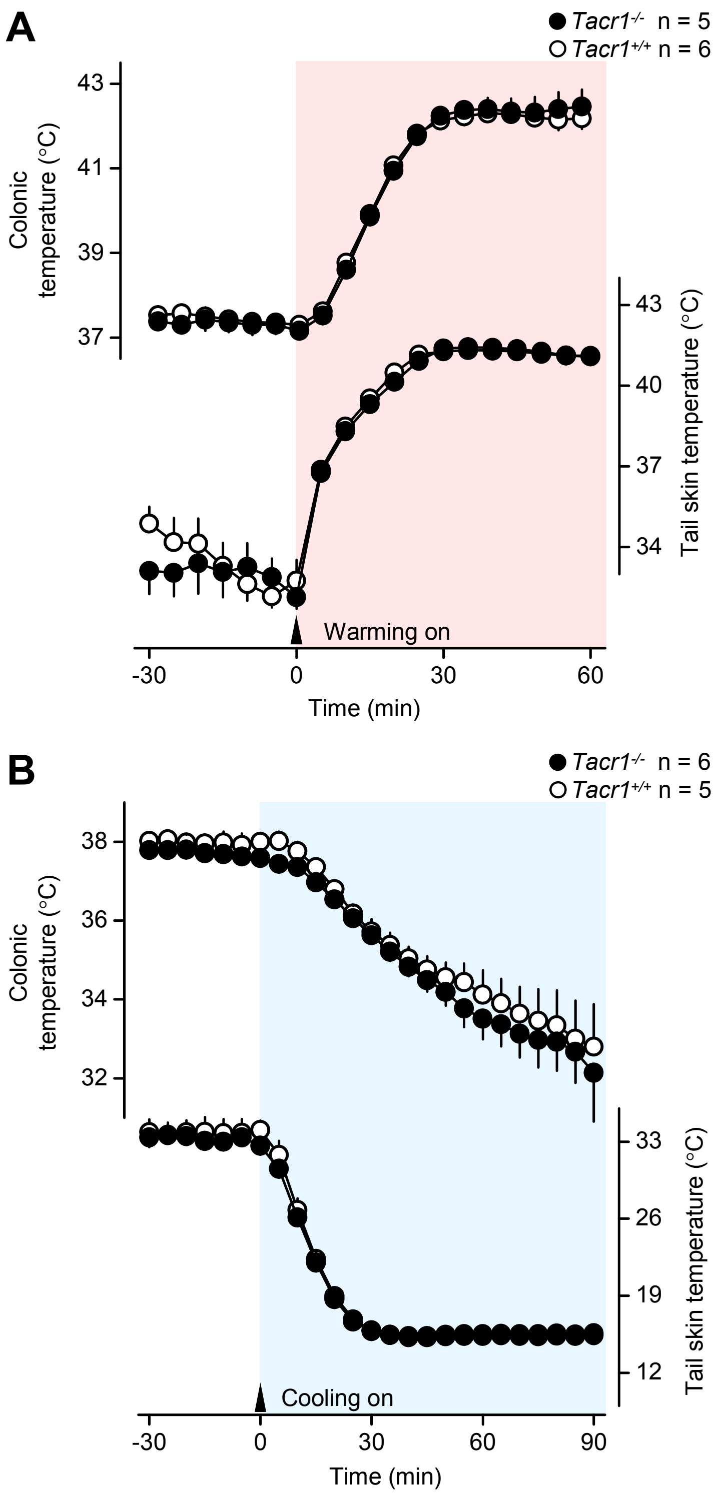

Supplementary figure 2.

The thermoregulatory response of *Tacr1*$^{+/+}$ and *Tacr1*$^{-/-}$ mice to thermal challenges. (**A**) Changes of colonic temperature and tail skin temperature in response to heat exposure in *Tacr1*$^{+/+}$ and *Tacr1*$^{-/-}$ mice. During heat exposure, ambient temperature was first raised from 33°C to 39°C at a rate of ~0.2°C/min and then maintained at 39°C until the end of the experiment. Colonic temperature and tail skin temperature responses of the two genotypes to heat exposure were identical. (**B**) Changes of colonic temperature and tail skin temperature in response to cold exposure in *Tacr1*$^{+/+}$ and *Tacr1*$^{-/-}$ mice. During cold exposure, ambient temperature was decreased from 33°C to 15°C at a mean rate of 0.6°C/min and then maintained at 15°C until the end of the experiment. Colonic temperature and tail skin temperature responses of the two genotypes to cold exposure were identical. Numbers of animals in the corresponding groups are indicated in the figure.
